# Courageous but Indebted? Regional Courage is Associated With Higher Debt-to-Income Ratio in the United States

**DOI:** 10.1177/01461672251398580

**Published:** 2025-12-29

**Authors:** Jali Packer, Joe Gladstone, Friedrich M. Götz

**Affiliations:** 1 Columbia University in the City of New York, NY, USA; 2 University of California San Diego, La Jolla, USA; 3 University of British Columbia, Vancouver, Canada

**Keywords:** courage, household debt, financial risk-taking, regional personality, geographical psychology

## Abstract

Geographic disparities in household indebtedness present an economic puzzle that traditional models inadequately explain. We examine whether regional psychological traits—specifically courage—help explain these differences. Analyzing data from 836,184 individuals across 1,220 U.S. counties, we tested whether areas with higher collective courage (willingness to act despite fear) exhibit higher debt-to-income ratios. Using spatial regression techniques to account for geographic clustering and controlling for sociodemographic factors and Big Five personality traits, we found that courage significantly predicted county-level debt-to-income ratios. A one standard deviation increase in regional courage was associated with a 0.22 standard deviation increase in debt-to-income—an effect that persisted across different geographic scales and modeling approaches. Courage hotspots in western and southern regions showed corresponding patterns of higher indebtedness. These findings reveal that psychological traits traditionally viewed as virtuous may have unintended economic consequences, highlighting the importance of considering regional psychology when designing financial policies and interventions.

Why do some regions of the United States consistently maintain higher household debt burdens than others? This question has significant implications given that U.S. household debt exceeds $18 trillion and exhibits persistent geographic patterns that defy complete explanation through traditional economic models (Board of Governors of the Federal Reserve System, 2024; Richter, 2020). These regional debt disparities matter profoundly: areas with elevated debt-to-income ratios experience increased financial vulnerability, including reduced consumer spending capacity, higher bankruptcy rates, and compromised health outcomes (Hershfield et al., 2015; Sweet et al., 2013). During economic downturns, heavily indebted regions suffer more severe contractions and recover more slowly, amplifying geographic inequality (Jordà et al., 2016; Mian & Sufi, 2010).

Although established economic predictors—including income distribution, educational attainment, and financial literacy—explain substantial variance in debt patterns (Gathergood et al., 2019; Lusardi & Mitchell, 2014), significant regional differences remain unaccounted for (Dynan et al., 2012). Recent work incorporating psychological dimensions into economic models has demonstrated how personality traits influence financial decision-making. For instance, conscientiousness typically predicts lower debt levels, while agreeableness correlates with higher borrowing (Brown & Taylor, 2014; Matz & Gladstone, 2020). However, these investigations have predominantly focused on the Big Five personality framework, potentially overlooking other relevant psychological traits. We propose that courage—defined as the willingness to act despite fear or perceived risk (Evans & White, 1981; O'Connell et al., 2024; Rachman, 2004)—represents a promising candidate for understanding regional variation in household debt.

## Courage as a Distinct Driver of Risk-Taking

Courage is defined as the tendency to act in the face of fear, risk, or potential adversity (Evans & White, 1981; O'Connell et al., 2024; Rachman, 2004), involving the conscious override of fear-driven avoidance in favor of approach-oriented behavior (Chockalingam & Norton, 2019; Cox et al., 1983). Unlike related constructs such as risk tolerance or sensation-seeking, courage specifically involves perceiving risk and experiencing fear but choosing to act regardless (Pury & Kowalski, 2007).

Historically conceptualized as a core virtue, courage has been studied primarily in contexts of moral behavior, leadership, and resilience (Dahlsgaard et al., 2005; Hannah et al., 2007). However, its role in financial decision-making remains underexamined despite compelling theoretical connections. Courageous individuals willingly face risk in pursuit of their goals, potentially driving more ambitious financial choices. For instance, those high in courage may be more likely to start businesses, invest in volatile assets, or assume substantial educational debt—actions that involve short-term costs for prospective long-term benefits.

Recent evidence supports this theoretical link: U.S. regions with higher aggregate courage demonstrate elevated rates of entrepreneurial activity (Ebert et al., 2019), suggesting that courageous communities foster cultures of boldness and initiative. However, this same research revealed that courage was simultaneously associated with higher rates of start-up failure, highlighting the double-edged nature of this trait. In personal finance, courage might motivate both productive investments (e.g., strategic educational loans) and imprudent borrowing (e.g., high-interest consumer debt), making it a particularly nuanced factor in understanding financial behavior.

## Regional Personality as a Framework for Understanding Economic Behavior

Geographical psychology provides a framework for investigating how aggregate psychological traits influence regional outcomes beyond individual-level effects (Götz et al., 2025; Rentfrow, 2010; Rentfrow et al., 2008). This approach reconceptualizes personality as operating at multiple levels of analysis, where regional traits emerge through selection processes (selective migration), socialization mechanisms (local cultural transmission), and institutional effects (formal and informal structures reflecting and reinforcing psychological tendencies). Regional traits thus represent meaningful constructs distinct from—though related to—individual personality differences (Ebert et al., 2021; Payne et al., 2017; Suliteanu et al., 2025).

Of direct relevance to the study at hand, regional personality traits predict substantial variance in important macro-level indicators after controlling for conventional economic and sociodemographic predictors. Evidence indicates robust associations between regional personality and economic outcomes (Ebert et al. 2022a). For instance, regions high in Openness enjoy higher average incomes and produce more high-impact innovations (Mewes et al., 2022; Rentfrow et al., 2015; Xu et al., 2026). Regions high in Agreeableness tend to exhibit lower income inequality but also face higher rates of personal insolvency and lower median incomes (Matz & Gladstone, 2020; Rentfrow et al., 2015). Meanwhile, regions high in Neuroticism are more vulnerable during economic crises (Obschonka et al., 2016). These effects operate through distinct mechanisms: compositional effects (the aggregation of individual-level associations) and contextual effects (where regional traits modify individual-level relationships). Importantly, multilevel analyses demonstrate that these contextual effects can alter behavior even among individuals who do not personally embody the regionally dominant trait (Ebert et al., 2021).

The relevance of regional courage for understanding household debt patterns stems from how financial decisions operate within normative environments that shape risk perception and borrowing behavior. While individual borrowing decisions reflect personal circumstances and characteristics (e.g., income, risk tolerance, financial literacy), these choices occur within cultural contexts that differentially frame debt’s meaning and acceptability. In regions characterized by high collective courage, prevailing narratives likely valorize financial boldness and resilience, potentially recasting debt as a calculated challenge to overcome rather than a burden to avoid. Conversely, low-courage regions may cultivate debt-averse norms wherein borrowing signals poor planning or financial inadequacy.

Importantly, regional psychological patterns themselves may partly reflect historical and structural forces. On the one hand, what manifests as “courage” in financial decision-making may be an adaptive response to systemic constraints rather than a purely dispositional trait. For example, in regions where traditional pathways to economic mobility are restricted, a greater willingness to assume financial risk might reflect necessity rather than true preference—a rational response to limited alternatives rather than an inherent tendency (Bertrand et al., 2004).

On the other hand, the spatial distribution of dispositional traits itself may be partly determined by such historical and structural forces. Indeed, previous research in geographical psychology has shown that regional personality traits are shaped by the historical legacy and contemporary structure of their respective area, including factors such as slavery (Payne et al., 2019), deindustrialization (Obschonka et al., 2018), frontier status (Götz et al., 2020b), war (Obschonka et al., 2017), and income inequality (Du et al., 2024). Taken together, this perspective suggests that observed regional variations in financial risk-taking may arise from both psychological dispositions and contextual adaptations to differential economic opportunities across geographic areas.

Regional courage presents a particularly compelling case for examining household debt patterns because financial decisions combine individual preferences with socially constructed risk perceptions. Recent advances in measuring regional courage (Ebert et al., 2019) enable us to test whether it systematically relates to financial behavior at the regional level. Critically, the psychological processes underlying courage (approach motivation despite perceived risk) parallel the psychological mechanisms involved in debt acquisition (accepting short-term costs for anticipated benefits despite uncertainty).

Analyzing courage at the regional rather than individual level offers several methodological advantages. First, it aligns the level of analysis with available economic data, which is typically aggregated geographically. Second, it addresses selection effects that confound individual-level financial research, as regional averages remain stable despite migration patterns (Elleman et al., 2018). Third, it captures emergent properties of social systems that cannot be reduced to individual traits, such as institutional norms and cultural narratives about debt (Calanchini et al., 2022; Hehman et al., 2019).

This regional approach aligns with a growing recognition in economic psychology that economic behaviors reflect both individual differences and social contexts (Ebert et al., 2021; Huggins & Thompson, 2017; Matz & Gladstone, 2020). By examining courage at the county level—a geographic unit that balances statistical power with meaningful cultural boundaries (cf. Ebert et al., 2023)—we can test whether this trait helps explain the persistent regional variation in household debt that economic models alone fail to account for.

## The Present Research

This study examines whether regional differences in courage—the collective willingness to act despite fear—help explain geographic variation in household debt burdens across the United States. Building on theoretical links between courage and financial risk-taking, we investigate whether counties characterized by higher aggregate courage exhibit systematically higher debt-to-income (DTI) ratios. We selected DTI rather than absolute debt levels because it provides a standardized measure of financial strain relative to economic resources, capturing households’ debt burden relative to their capacity for repayment (Ahn et al., 2018; Board of Governors of the Federal Reserve System, 2024). This allows for meaningful comparisons across regions with different income distributions.

Our investigation extends previous research on regional courage in two important ways. First, whereas Ebert et al. (2019) primarily examined courage as a predictor of entrepreneurial activity, we focus specifically on household financial behavior. Second, while their supplementary analyses reported a bivariate correlation between courage and consumer debt, we conduct comprehensive multivariate analyses that account for socioeconomic factors, other personality dimensions, and spatial dependencies—with a sample more than twice as large. This allows us to isolate courage’s unique contribution to regional debt patterns beyond established demographic, economic predictors, and broadband personality traits.

We test our hypothesis at the county level for several reasons. Counties represent stable administrative units with consistent data availability across multiple domains (economic, demographic, and psychological). This geographic scale balances granularity with statistical reliability—counties are sufficiently localized to capture meaningful cultural variation yet large enough to yield stable personality estimates with adequate sample sizes (Ebert et al., 2023; Matz & Gladstone, 2020). The county level also aligns with the implementation scale of many financial policies (Garretsen & Stoker, 2023; Peters et al., 2023), enhancing the practical relevance of our findings.

Specifically, we hypothesize that after controlling for sociodemographic factors (income, age, education, unemployment, poverty, and population density) and established personality traits (Openness, Conscientiousness, Extraversion, Agreeableness, and Neuroticism), counties with higher aggregate courage scores will exhibit significantly higher DTI ratios (H1). We further predict this relationship will remain robust when accounting for spatial autocorrelation through appropriate spatial regression techniques (H2).

## Method

### Participants and Procedure

We analyzed personality data from the TIME Magazine Sorting Hat Project (Götz et al., 2020a), a large-scale internet-based survey conducted between June 2017 and February 2024 (*N* = 2,344,106). This project received ethical approval from the University of Cambridge Psychology Research Ethics Committee (PRE.2017.044). Participants were recruited through TIME Magazine’s digital platforms and affiliated outlets, including *People* and *Entertainment Weekly*. The survey consisted of 21 questions (see Supplemental Files – Table S1) measuring various personality dimensions, including Courage, Conscientiousness, and Machiavellianism. An automated matching algorithm then used participants’ responses to assign them to a fictional ‘Hogwarts House’ from the Harry Potter series (for details see Götz et al., 2020a).

We restricted our analyses to respondents from the United States aged 18–65 with complete survey responses and valid ZIP codes, yielding a sample of 895,657 participants (70% female; *M*_age_ = 30.67 years, *SD* = 9.78). The sample’s racial/ethnic composition was 74% White, 8% Asian, 8% Hispanic, 4% Black, and 6% Other.^1^ The sample included participants from all 50 states and the District of Columbia, indicating broad geographic coverage.

### Geographic Aggregation Procedure

Each participant’s personality data was linked to their residential county via ZIP Code Tabulation Area (ZCTA) crosswalks (U.S. Census Bureau, 2010). Following methodological standards in geographical psychology (Ebert et al., 2022b; Matz & Gladstone, 2020), we established minimum sample requirements, including only counties with at least 50 respondents after data cleaning. This conservative threshold balanced comprehensive geographic coverage with statistical reliability, resulting in a final analytical sample of 1,220 counties (*N* = 836,184), representing 39% of all U.S. counties but covering 89% of the U.S. population. The sample size across counties varied (*M_N_* = 685, SD = 1,801), ranging from 50 in the smallest counties to 35,393 in Los Angeles County.

### Measures

#### Courage

Regional courage was measured using the 6-item Courage Measure (Howard & Alipour, 2014; Norton & Weiss, 2009). Items included statements such as “I tend to face my fears” and “Even if something scares me, I will not back down,” rated on 7-point scales (1 = “Strongly disagree” to 7 = “Strongly agree”). The scale demonstrated strong internal consistency (α = .82) and meaningful between-county variation (ICC_2_ = .75), supporting its suitability for our analyses. The average courage score across counties was M = 4.76 (SD = 0.13) on the 1–7 scale.

#### Debt-to-Income Ratio

County-level DTI ratios were calculated as total household debt divided by total household income, providing a standardized measure of debt burden. Data were sourced from the Federal Reserve’s Enhanced Financial Accounts database (Board of Governors of the Federal Reserve System, 2024), matched to the 2022 calendar year, aligning with the central period of our personality data collection. The mean DTI ratio across counties was 1.82 (SD = 0.79), indicating that, on average, households carried debt approximately 1.8 times their annual income.

#### Additional Personality Traits

To establish the discriminant validity of courage as a predictor, we included the Big Five personality traits (Openness, Conscientiousness, Extraversion, Agreeableness, and Neuroticism) as controls. We sourced these data from the Gosling-Potter Internet Personality Project (Gosling et al., 2004), using county-level aggregates reported by Peters et al. (2023). This allowed us to isolate the unique contribution of courage beyond established personality dimensions known to influence economic behavior.

#### Controls

Following standard practices in geographical psychology (Ebert et al., 2022a; Götz et al., 2020b; Rentfrow, 2010), we included county-level demographic and economic indicators: median household income, mean age, gender composition (percent female), unemployment rate, educational attainment (percent with bachelor’s degree or higher), poverty rate (percent below federal poverty line), and population density (residents per square mile). These data were obtained from the U.S. Census Bureau (2022) and the Economic Research Service (2023).

#### Standardization

All variables were *z*-score standardized to have a mean of 0 and a standard deviation of 1, which enabled direct comparisons of effect sizes.

### Transparency and Openness

This research was not preregistered. To support scientific transparency and reproducibility, we have made all analysis code, derived variables, spatial weights matrices, and data publicly available through our OSF repository (https://osf.io/afbs8/).

### Analytic Approach

#### Quantifying and Visualizing Spatial Clustering

To evaluate whether courage and DTI ratios cluster geographically, we conducted formal tests of spatial autocorrelation. Using Moran’s I statistic (Cliff & Ord, 1981; Moran, 1950), we quantified the degree to which similar values appear in geographic proximity. This analysis required constructing a spatial weights matrix that formally defines “neighboring” relationships between counties. Following methodological standards in spatial econometrics (Obschonka et al., 2019), we employed a distance-based criterion that defined neighbors as counties with centroids within 100km. For geographically isolated counties, we assigned their nearest county as a neighbor (e.g., Matz & Gladstone, 2020). To visualize the spatial clustering, we employed Getis-Ord Gi* hotspot analysis (Getis & Ord, 1992; Ord & Getis, 1995), which identifies statistically significant clusters of high values (hotspots) and low values (coldspots) relative to the national distribution. Unlike simple choropleth maps that display raw values, this technique incorporates spatial weights and statistical significance testing, producing standardized *z*-scores that quantify clustering intensity while controlling for random spatial variation.

#### Regression Analysis and Robustness Checks

Our analysis proceeded in three stages. First, we estimated ordinary least squares (OLS) regression models at the county level: a baseline model with courage as the sole predictor of DTI ratios, and a comprehensive model including all personality and socioeconomic controls. Second, recognizing that geographic units often violate the independence assumption of standard OLS regression, we employed spatial regression techniques to account for spatial autocorrelation (Anselin, 1988; Ebert et al., 2023). Third, to address the modifiable areal unit problem (MAUP; Fotheringham & Wong, 1991), we replicated our analyses at broader geographic scales—Metropolitan Statistical Areas (MSAs) and states—testing whether the courage-debt relationship persisted regardless of spatial aggregation level. County-level results are presented here, and the parallel MSA- and state-level results are reported in the Supplemental Files.

## Results

### Spatial Clustering of Courage and Debt Across the United States

County-level courage exhibited substantial spatial autocorrelation (Moran’s *I* = 0.26, *p* < .001). This finding indicates that counties with above-average courage scores are significantly more likely to be surrounded by other high-courage counties, while low-courage counties similarly cluster together. This is a typical pattern in geographical research, so common, in fact, that it became enshrined as Tobler’s first law of geography: “*Everything is related to everything else, but near things are more related than distant things*.” (Tobler, 1970, p. 236). DTI ratios showed moderate but significant spatial dependence (Moran’s *I* = 0.12, *p* < .001), confirming that financial behaviors also display nonrandom geographic patterning.

Figure 1 reveals distinct regional patterns in courage across the United States. Statistically significant courage hotspots (counties with *z*-scores > 1.96, *p* < .05) form distinct clusters in the Western and Southern regions. Particularly strong hotspots (*z*-scores > 2.58, *p* < .01) appear in California, the Pacific Northwest, the Mountain West (Utah, Wyoming, Colorado), and across the South, with notable concentrations in Texas, Oklahoma, and Florida. In contrast, significant courage coldspots (*z*-scores < −1.96, *p* < .05) dominate the Northeast and Upper Midwest, creating an extensive pattern throughout New England, New York, Pennsylvania, Michigan, Wisconsin, Minnesota, and Iowa. This geographic distribution reveals a notable East-West divide in regional courage profiles, though with substantial within-region heterogeneity rather than a simple gradient. The pattern demonstrates remarkable consistency with previous findings at the metropolitan level (Ebert et al., 2019), suggesting that regional courage maintains stable geographic distributions across different spatial aggregation levels—a key validation of the regional personality construct.

**Figure 1.**
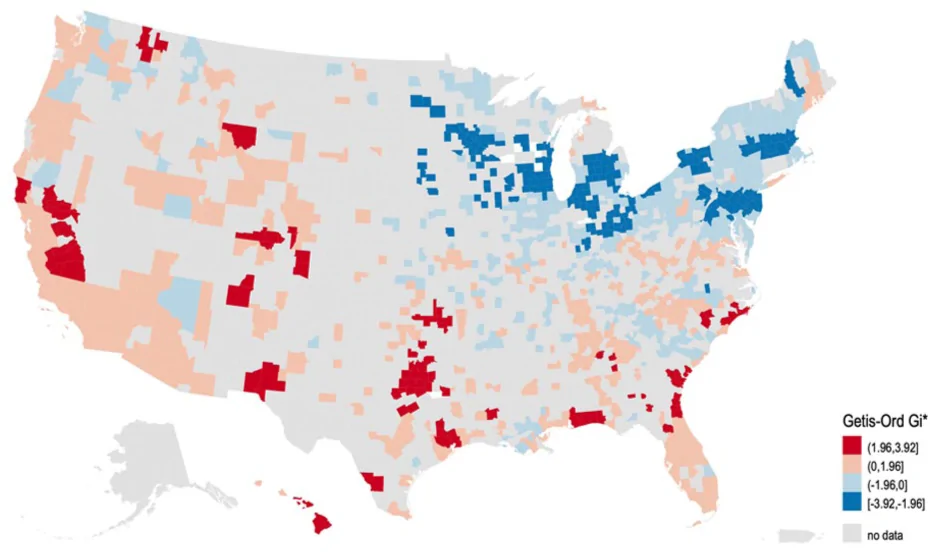
Hotspot analysis showing regional variation of courage across the United States (county level). *Note*. Shades of red indicate hotspots (i.e., clusters of areas with high levels of courage), whereas shades of blue indicate coldspots (i.e., clusters of areas with low levels of courage). *N*_county_ = 1,220.

Figure 2 displays the spatial distribution of household DTI ratios, revealing patterns that visually correspond to the courage distribution. High-DTI hotspots (*z*-scores > 1.96, *p* < .05) appear prominently across the Southeast (particularly in Georgia, South Carolina, and Florida) and in parts of the Western United States (Nevada, Arizona, and California). In contrast, significantly low-DTI coldspots (*z*-scores < −1.96, *p* < .05) are concentrated in New England, the Upper Midwest, and Northern Plains states.

**Figure 2.**
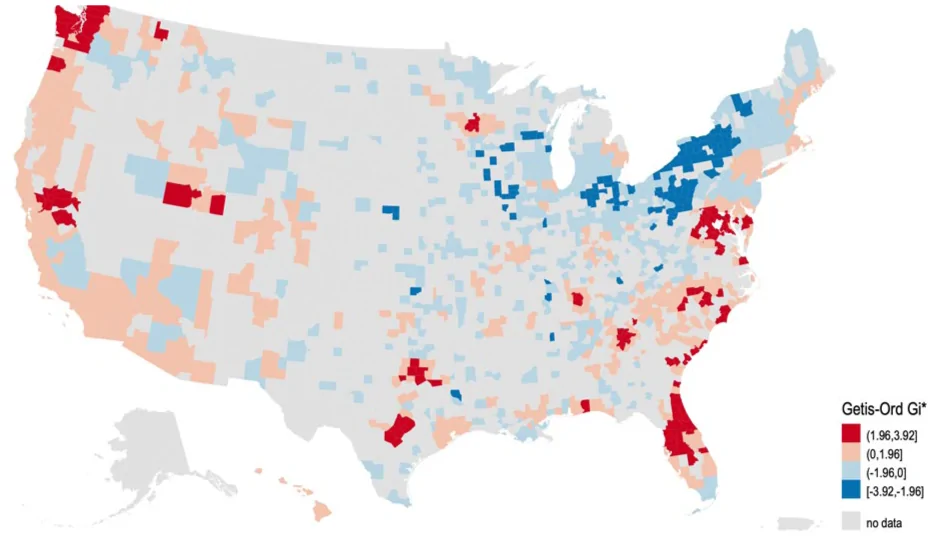
Hotspot analysis showing regional variation of debt-to-income ratio across the United States (county level). *Note*. Shades of red indicate hotspots (i.e., clusters of areas with high levels of debt-to-income), whereas shades of blue indicate coldspots (i.e., clusters of areas with low levels of debt-to-income). *N*_county_ = 1,220.

Figure 3 combines Figures 1 and 2, displaying the spatial distribution of household courage and household DTI ratios concurrently in a bivariate choropleth map. Figure 3 shows that high-courage, high-DTI hotspots are predominantly located in Georgia, Florida, California, and the Pacific Northwest. Analogously, we can see patterns of low-courage and low-DTI coldspots, most notably spanning from the Great Lakes region to the Middle Atlantic states and New England. These areas of convergence are frequent, while areas of divergence (high Courage and low DTI or low Courage and high DTI) are less common. Notable areas of divergence include high-courage but low-DTI pockets along the Appalachian Mountains, and low-courage but high-DTI areas in places like Minnesota and Maryland.

**Figure 3.**
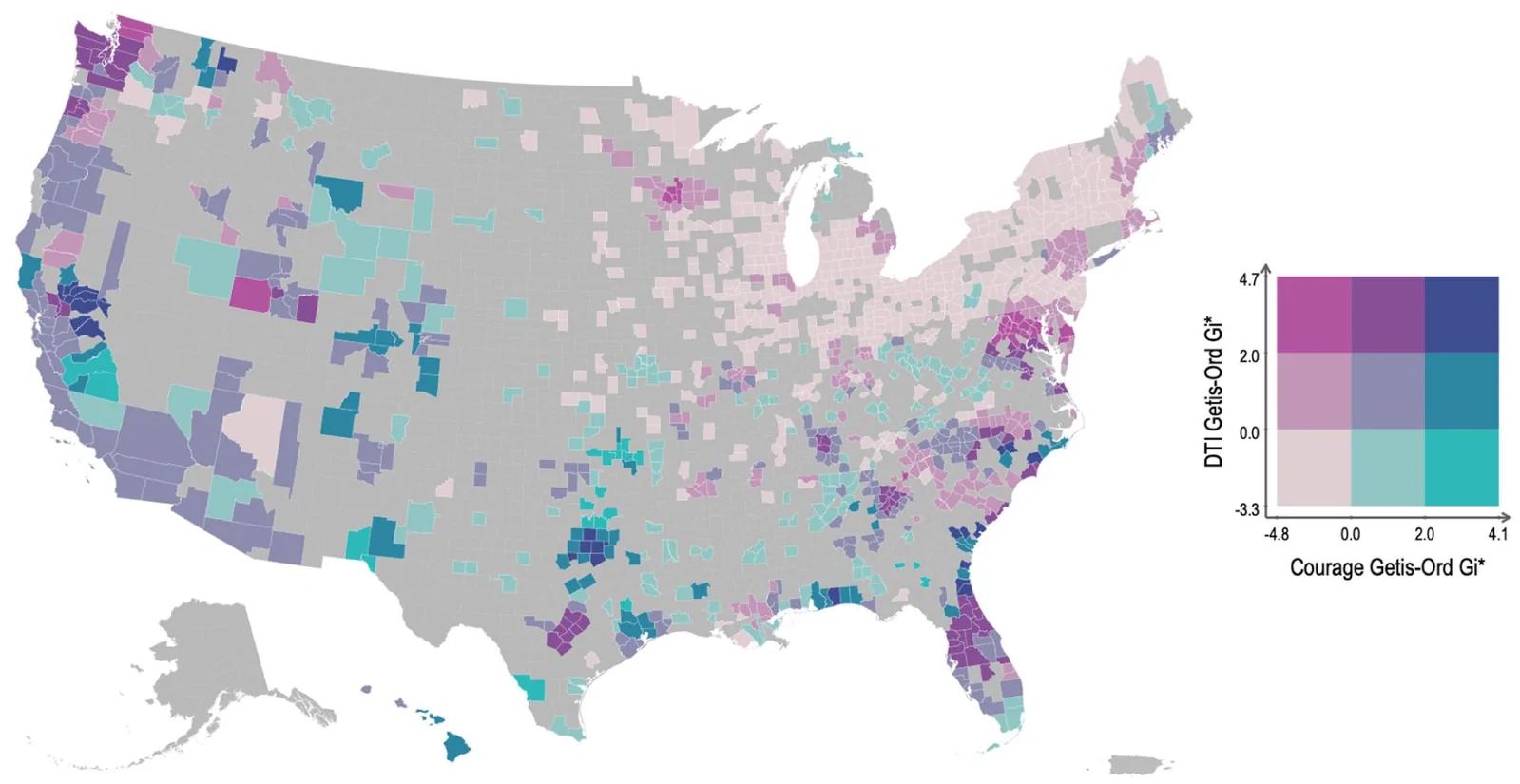
Combined hotspot analysis showing regional variation of courage and debt-to-income ratio across the United States (county level). *Note*. Shades of dark blue indicate high debt-to-income and high-courage hotspots (i.e., clusters of areas with both high levels of debt-to-income and high levels of courage). Shades of dark green indicate high levels of courage but low levels of debt-to-income, whereas shades of dark purple indicate high levels of debt-to-income but low levels of courage. *N*_county_ = 1,220.

### Bivariate and Multivariate Regression Analyses

Consistent with our primary hypothesis (i.e., H1: counties with higher aggregate courage scores will exhibit significantly higher DTI ratios), OLS regression revealed that county-level courage was significantly positively associated with DTI ratios (Table 1). In the baseline model with courage as the sole predictor, higher regional courage corresponded to higher debt burden (β = .22, *SE* = .028, *t*(1218) = 7.85, *p* < .001). This standardized coefficient indicates that a one standard deviation increase in regional courage was associated with a 0.22 standard deviation increase in DTI ratios. To contextualize this effect: a 1-point increase on the 7-point courage scale corresponded to a 1.28-point increase in DTI ratios—a substantial effect considering these ratios ranged from 0.78 to 3.43 across counties.

**Table 1.** OLS Regression Predicting County Debt-to-Income Ratio From Courage (Model 1a) and Courage with Controls (Model 1b).

	1a Courage			1b Courage + Controls		
Predictor	*B*	*SE* (B)	*t*	*B*	*SE* (B)	*t*
Predictor						
Courage	.22***	.028	7.85	.24***	.025	9.85
Controls						
Openness	—	—	—	−.03	.032	−.86
Agreeableness	—	—	—	.06	.036	1.57
Extraversion	—	—	—	−.07*	.028	−2.43
Conscientiousness	—	—	—	.03	.032	1.03
Neuroticism	—	—	—	−.11**	.035	−3.15
Income	—	—	—	.40***	.050	7.92
Age	—	—	—	.18***	.029	6.32
Gender	—	—	—	.014	.026	.53
Unemployment	—	—	—	.02	.027	.57
Poverty	—	—	—	−.26***	.048	−5.41
Population density	—	—	—	−.05*	.025	−2.09
Education	—	—	—	−.29***	.042	−7.03
*R*^2^	.05			.36		

*Note. N* = 1,220. All variables were *z*-standardized.

* *p* <* .05.* **p <* .01.* ***p <* .001.*

This relationship not only persisted but strengthened slightly when accounting for demographic and personality covariates (β = .24, *SE* = .025, *t*(1,206) = 9.85, *p* < .001). The modest increase in effect size indicates a suppression effect (Paulhus et al., 2004), suggesting that certain control variables (particularly income and education) were masking part of the courage-debt relationship in the simpler model. The full model explained substantial variance in county-level debt burden (*R2* = .36), with courage remaining one of the strongest predictors alongside economic factors.

Several control variables showed significant associations with DTI ratios, providing additional insight into regional debt patterns. Higher income counties (β = .40, *p* < .001) and those with older populations (β = .18, *p* < .001) exhibited significantly higher debt burdens, consistent with greater credit access and longer credit histories. Conversely, counties with higher poverty rates (β = −.26, *p* < .001) and higher educational attainment (β = −.29, *p* < .001) showed significantly lower DTI ratios, likely reflecting limited credit access in economically disadvantaged areas and more conservative financial practices in more educated regions.

Among Big Five traits, only Neuroticism (β = −.11, *p* = .002) and Extraversion (β = −.07, *p* = .015) significantly predicted regional debt burden, and both were negative predictors. Moreover, their effects were much smaller than the effect of courage—approximately half the size for Neuroticism and about one-third for Extraversion. Notably, neither Con-scientiousness nor Agreeableness—traits previously linked to individual-level financial behavior (Brown & Taylor, 2014; Matz & Gladstone, 2020)—significantly predicted county-level debt when controlling for courage and socioeconomic factors. This pattern underscores courage’s unique contribution to understanding regional financial behavior beyond established personality dimensions.

### Spatial Regressions: Courage Predicts Debt Beyond Geographic Clustering

We next employed spatial lag regression models, which explicitly account for geographic dependencies by incorporating the weighted average of neighboring counties’ DTI ratios as a predictor variable (Anselin, 1988; Ebert et al., 2023). This approach addresses potential bias from spatial spillover effects—where economic conditions, lending practices, and financial norms may transcend administrative boundaries. Table 2 presents the results of these spatial regression analyses.

**Table 2.** Spatial Lag Regression Predicting County Debt-to-Income Ratio from Courage (Model 2a) and Courage with Controls (Model 2b).

	2a Courage			2b Courage + Controls		
Predictor	*B*	*SE* (*B*)	*z*	*B*	*SE* (*B*)	*z*
Predictor						
Courage	.20***	.028	7.06	.23***	.024	9.42
Controls						
Openness	—	—	—	–.05	.032	–1.50
Agreeableness	—	—	—	.05	.035	1.53
Extraversion	—	—	—	–.06*	.027	–2.07
Conscientiousness	—	—	—	.03	.032	.94
Neuroticism	—	—	—	–.09**	.035	–2.60
Income	—	—	—	.38***	.049	7.72
Age	—	—	—	.18***	.028	6.41
Gender	—	—	—	.01	.026	.22
Unemployment	—	—	—	.01	.027	.54
Poverty	—	—	—	–.26***	.047	–5.46
Population density	—	—	—	–.04	.025	–1.78
Education	—	—	—	–.28***	.04	–6.90
*R* ^2^	.05			.36		

*Note. N* = 1,220.

Spatial lag models include a term (ρ) for spatially lagged dependent variable influence. All variables were z-standardized. *p < .05. **p < .01. ***p < .001. To check for spatial autocorrelation of error terms after the spatial lag correction, a postestimation Moran’s test for spatial dependency was conducted returning a nonsignificant result (χ = 0.15; *p* = .70).

In the baseline spatial model, courage remained a significant predictor of DTI ratios (*β* = .20, *SE* = 0.028, *z* = 7.06, *p* < .001), with only a slight reduction in coefficient magnitude compared to the OLS estimate (0.20 vs. 0.22). In the comprehensive model with socioeconomic and personality controls, courage’s predictive strength actually increased slightly (*β* = 0.23, *SE* = 0.024, *z* = 9.42, *p* < .001). This pattern demonstrates that the courage-debt relationship is not merely an artifact of spatial clustering or regional contagion effects.

The spatial lag parameter (*ρ* = 0.35, *p* < .001) revealed substantial geographic dependency, indicating that approximately 35% of the variation in a county’s DTI ratio could be attributed to debt levels in neighboring counties. This significant spillover effect underscores the importance of modeling spatial processes explicitly. Importantly, courage’s predictive power remained robust even after accounting for these spatial dependencies. Postestimation diagnostics confirmed the spatial model’s effectiveness, with residual spatial autocorrelation reduced to non-significance (Moran’s I = 0.01, *p* = .70).

### Cross-Scale Replication: Testing Robustness Across Geographic Units

A persistent challenge in geographical analysis is the MAUP—the possibility that results might depend on the specific geographic boundaries used (Fotheringham & Wong, 1991; Manley, 2014). To address this concern, we conducted parallel analyses at multiple geographic scales to determine whether the courage-debt relationship remained consistent regardless of spatial aggregation level (for full results, see Tables S3–S6 in the Supplemental Files).

At the MSA level, courage significantly predicted DTI ratios in both our baseline model (*β* = .26, *SE* = 0.049, *z* = 5.25, *p* < .001) and comprehensive model with controls (*β* = .24, *SE* = 0.051, *z* = 4.69, *p* < .001). The consistency in effect sizes between county-level (0.20–0.23) and MSA-level analyses (0.24–0.26) demonstrates remarkable stability across different spatial resolutions.

State-level analyses showed a larger effect in the baseline model (*β* = .46, *SE* = 0.137, *z* = 3.37, *p* < .01) and a positive but nonsignificant association in the full model (*β* = 0.20, *SE* = 0.142, *z* = 1.40, *p* = .16). The attenuated significance likely reflects reduced statistical power at the state level (*N* = 49) rather than a substantive difference in the relationship (cf. Rentfrow et al., 2008).

## General Discussion

Our findings reveal that regions characterized by higher collective courage tend to carry heavier household debt burdens, even after accounting for socioeconomic factors, Big Five personality traits, and spatial dependencies. This pattern suggests that psychological characteristics traditionally viewed as virtues may have complex economic implications that vary across contexts.

These results extend current understanding in several ways. First, they demonstrate the value of examining specific psychological constructs beyond the dominant Big Five framework when analyzing regional economic behaviors (Mõttus et al., 2020). While previous research has linked traits such as Conscientiousness and Agreeableness to individual financial outcomes (Brown & Taylor, 2014; Matz & Gladstone, 2020), our findings indicate that courage provides unique explanatory power for understanding regional variation in financial behavior. While the Big Five remains a highly effective framework for capturing personality in broad strokes (Bainbridge et al., 2022), our findings suggest that extending geographical psychology to include narrower, more targeted traits can yield important new insights at the regional level.

Second, our work bridges personality psychology with economic sociology and cultural psychology perspectives. The association between regional courage and debt patterns reveals how shared psychological tendencies shape economic behaviors through both compositional mechanisms (aggregated individual dispositions) and contextual influences (regional norms). In high-courage regions, the cultural climate may normalize financial risk-taking and frame debt as an acceptable tool for pursuing opportunities. These regional differences may reflect historically rooted cultural patterns. For instance, the American South’s combination of high courage and high debt might stem from a culture of honor and boldness (Nisbett & Cohen, 1996) that values decisive action while downplaying financial caution. Conversely, New England’s profile of lower courage and lower debt aligns with a traditionally cautious regional culture, perhaps reflecting the legacy of Puritan values in that region. This perspective complements research showing how regional psychological characteristics create environments that amplify, modify, or override individual-level personality effects (Ebert et al., 2021; Stavrova, 2015).

Third, these findings suggest a more nuanced understanding of courage itself. Rather than representing an unambiguous virtue, courage functions as a double-edged sword in the economic domain—potentially promoting both productive investments and excessive financial risk-taking. This duality parallels previous work showing courage’s association with both higher rates of business formation and business failure (Ebert et al., 2019), highlighting how traits beneficial in some contexts may create vulnerabilities in others (Nettle, 2006). While our framework assumes that regional courage influences debt patterns, alternative explanations should be considered—for example, regional economic conditions might shape the development or expression of collective courage, rather than the other way around.

## Structural Context and Historical Legacy

The observed geographic patterns should be considered alongside historical and contemporary structural factors that may influence regional economic behavior. Historical and contemporary structural forces may influence economic behavior indirectly by shaping regional personality traits, as shown in prior research (Du et al., 2024; Götz et al., 2020b; Obschonka et al., 2017, 2018; Payne et al., 2019). If that is the case, then their impact would already be accounted for in our models (which include regional personality indicators).

On the other hand, historical and contemporary structural forces may also exert a direct impact on regional economic behavior. To illustrate, the correlation between high courage and high debt in Southern regions occurs within a context of historical policies that differentially affected access to financial resources across demographic groups (Baradaran, 2017; Rothstein, 2017). These historical differences in credit access and wealth accumulation opportunities may have contributed to the development of region-specific financial strategies and norms that persist in contemporary patterns.

In addition, current structural variations across regions—including differences in financial institution density, availability of alternative financial services, and implementation of consumer protection regulations—may shape the contexts within which financial decisions occur (Friedline & Chen, 2021). The construct we identify as psychological “courage” may therefore reflect both dispositional traits and learned behavioral responses to regional economic conditions. In areas with restricted access to traditional financial services, observed risk tolerance could represent an adaptive strategy developed in response to environmental constraints rather than solely an individual difference variable. This interpretation underscores the importance of considering both psychological and structural factors when examining regional variation in financial behavior. Indeed, our models controlled for several structural indicators—such as regional poverty, unemployment, and educational attainment—to better isolate the unique effect of regional courage.

## Implications for Policy and Practice

Our findings suggest that financial interventions should be calibrated to regional psychological profiles rather than applying uniform approaches across diverse populations. In high-courage regions, where over-leveraging appears more prevalent, financial education initiatives might emphasize realistic risk assessment and prudent debt management—tempering optimistic biases with concrete illustrations of long-term interest costs and repayment scenarios. Conversely, in regions characterized by lower courage, programs could productively encourage responsible risk-taking to avoid overcautiousness that might stifle economic activity. Recent experimental research supports this targeted approach, demonstrating that financial education shows greater effectiveness when aligned with participants’ psychological characteristics (Matz et al., 2023). The substantial geographic variation in courage we documented suggests that regionalizing intervention content could significantly enhance program efficacy.

Financial institutions could similarly incorporate regional psychological profiles into their practices. Lenders operating in high-courage areas might develop specialized products that include built-in safeguards against excessive borrowing or offer enhanced financial planning support alongside credit products. Consumer protection agencies could benefit from regionally calibrated oversight strategies, directing greater scrutiny to areas where psychological tendencies toward financial risk-taking create vulnerability to predatory practices. These approaches would refine existing geographic targeting methods that typically rely exclusively on economic indicators rather than psychological characteristics.

Perhaps most consequentially, our findings inform understanding of regional economic resilience and crisis response. Prior research shows that areas with high DTI ratios experience more severe contractions during recessions (Jordà et al., 2016), with slower recoveries and sharper declines in consumption and employment (Mian et al., 2013; Verner & Gyöngyösi, 2020). Our results suggest that high-courage regions, with their correspondingly higher debt burdens, may be systematically more vulnerable to economic shocks—a critical consideration for macroprudential policy. Paradoxically, the same psychological characteristics that increase vulnerability during downturns might accelerate recovery through increased entrepreneurship and economic initiative. This duality suggests that targeted policy approaches—combining debt-relief programs with support for entrepreneurial activities in high-courage regions—could both mitigate vulnerabilities and leverage regional psychological strengths for more effective crisis response. By accounting for how regional psychological characteristics shape economic behavior patterns, policymakers can develop more nuanced approaches that enhance financial resilience while respecting regional differences in risk orientation.

## Limitations and Future Directions

The cross-sectional nature of our data represents the most significant limitation, precluding causal inference despite the robust associations observed (Shadish et al., 2002). Although theoretical arguments favor courage influencing debt patterns, alternative causal directions remain plausible—regional economic conditions might shape psychological dispositions, or unmeasured third variables could influence both. Future research could address this limitation through time-lagged designs that track regional courage and DTI trajectories over multiple measurement points (e.g., Götz et al., 2021) or through quasi-experimental approaches leveraging exogenous policy changes that affect debt accessibility across regions with varying courage profiles (Angrist & Pischke, 2009). Natural experiments, such as the staggered implementation of financial regulations across states, could provide particularly valuable causal insights.

Our reliance on self-reported courage introduces measurement concerns related to social desirability, response styles, and reference-group effects (Podsakoff & Organ, 1986; Schwarz, 1999). While the courage scale demonstrates strong psychometric properties (Howard & Alipour, 2014) and cross-region comparison biases are attenuated in within-country studies (Heine & Buchtel, 2009), future research would benefit from triangulating self-reports with behavioral measures of risk-taking, informant ratings from community members, or objective indicators from administrative data. In addition, although DTI ratios provide a standardized metric for cross-region comparison, this measure cannot distinguish between productive debt (e.g., education loans, mortgages) and potentially problematic consumer debt—a distinction that future studies should incorporate to refine understanding of courage’s financial implications.

Sampling considerations present another limitation. Our personality data derive from a nonrandom internet sample with demographic distributions that differ from the general U.S. population. While substantial evidence indicates that large convenience samples can yield valid geographic personality estimates when aggregated (Ebert et al., 2022a; Rentfrow et al., 2008), and our sample showed a strong correlation between participant numbers and regional population distribution (*r* = .95; Ebert et al., 2019), future research would benefit from more representative sampling approaches. The county-level analysis threshold (minimum 50 respondents) also excluded approximately 61% of U.S. counties, potentially limiting generalizability to more rural regions.

The U.S.-specific nature of our investigation limits cross-cultural generalizability. Cultural attitudes toward debt vary substantially across nations (Goetzmann & Rouwenhorst, 2005), with some countries demonstrating strong debt aversion (e.g., Germany; Bagnall et al., 2016) that might moderate the relationship with borrowing. Cross-national studies examining how courage relates to debt patterns across diverse economic systems and cultural contexts would significantly extend theoretical understanding of when and how psychological traits influence financial behavior.

A notable limitation concerns the absence of direct measures for several potentially relevant structural variables, including historical lending patterns, financial institution accessibility, and intergenerational wealth transmission. Consequently, the courage construct measured here may capture both dispositional characteristics and behavioral adaptations to economic constraints, which cannot be disentangled in the current analysis. Future investigations would benefit from incorporating indicators of structural economic factors, such as regional banking density, historical credit access patterns, and wealth distribution metrics. Such variables could be examined as potential moderators of the courage-debt relationship to determine whether psychological associations vary systematically across different structural contexts. Multilevel modeling approaches that simultaneously examine individual psychological characteristics, regional psychological aggregates, and structural economic indicators would yield a more comprehensive understanding of the factors contributing to geographic variation in household debt patterns. In addition, as suggested above, longitudinal designs tracking changes in both regional psychological profiles and structural economic conditions over time would help clarify the directionality and relative contributions of these different influences.

Finally, future work should expand beyond courage to examine interactions among multiple psychological traits that jointly influence regional financial outcomes. Dimensions such as future time perspective, delay discounting, impulsivity, and sensation-seeking likely operate alongside courage to shape complex financial behaviors. Multi-trait approaches could develop more comprehensive psychological profiles of regions (cf., Rentfrow et al., 2013), potentially identifying distinct regional “types” with characteristic financial vulnerabilities and strengths. Such research would advance understanding of how collective personality shapes economic trajectories and inform increasingly targeted policy interventions to promote financial well-being across psychologically diverse regions.

## Conclusion

Our findings add to the growing body of work on regional personality and economic outcomes, highlighting courage as a significant psychological factor in U.S. households’ financial decision-making patterns. Overall, our results call for a more psychologically informed approach to regional economic disparities and suggest that interventions addressing financial behavior should account for the distinctive psychological profiles of different communities.

## Supplemental Material

sj-docx-1-psp-10.1177_01461672251398580 – Supplemental material for Courageous but Indebted? Regional Courage is Associated With Higher Debt-to-Income Ratio in the United States Supplemental material, sj-docx-1-psp-10.1177_01461672251398580 for Courageous but Indebted? Regional Courage is Associated With Higher Debt-to-Income Ratio in the United States by Jali Packer, Joe Gladstone and Friedrich M. Götz in Personality and Social Psychology Bulletin

## Notes

1. The sample’s racial composition (74% White) exceeds the U.S. population proportion (61.6% White; U.S. Census Bureau, 2021), potentially limiting generalizability to communities with different demographic profiles and debt experiences, particularly those disproportionately affected by structural economic inequalities.
