# Supplementary material for: Courageous but Indebted? Regional Courage is Associated With Higher Debt-to-Income Ratio in the United States
Source: Pers Soc Psychol Bull. 2025 Dec 29;52(12):4154–66. doi: 10.1177/01461672251398580 (PMC13736281; doi:10.1177/01461672251398580)
Supplement: Supplementary file 1 [file sj-docx-1-psp-10.1177_01461672251398580.docx]

**Supplementary Files**

**Table S1.** *Survey questions used for the TIME Magazine Sorting Hat Dataset, traits assessed and source.*

| **Question** | **Trait assessed** | **Source** |
| --- | --- | --- |
| I see myself as critical, quarrelsome. (r) | Agreeableness | Gosling et al., 2003 |
| I see myself as sympathetic, warm | Agreeableness | Gosling et al., 2003 |
| I see myself as dependable, self-disciplined. | Conscientiousness | Gosling et al., 2003 |
| I see myself as disorganized, careless. (r) | Conscientiousness | Gosling et al., 2003 |
| When working, I often set ambitious goals for myself. | Conscientiousness | Gosling et al., 2003 |
| I often push myself very hard when trying to achieve a goal. | Conscientiousness | Gosling et al., 2003 |
| I do only the minimum amount of work needed to get by. (r) | Conscientiousness | Gosling et al., 2003 |
| I often check my work over repeatedly to find any mistakes (r) | Conscientiousness | Gosling et al., 2003 |
| I tend to face my fears. | Courage | Howard & Alipour, 2014 |
| Even if I feel terrified, I will stay in the situation until I have done what I need to do. | Courage | Howard & Alipour, 2014 |
| I will do things even though they seem to be dangerous | Courage | Howard & Alipour, 2014 |
| If I am worried or anxious about something, I will do or face it anyway | Courage | Howard & Alipour, 2014 |
| If there is an important reason to face something that scares me, I will face it | Courage | Howard & Alipour, 2014 |
| Even if something scares me, I will not back down. | Courage | Howard & Alipour, 2014 |
| I am an ordinary person who is no better than others. | Honesty-Humility | Lee & Ashton, 2018 |
| I wouldn't want people to treat me as though I were superior to them. | Honesty-Humility | Lee & Ashton, 2018 |
| I think that I am entitled to more respect than the average person is. (r) | Honesty-Humility | Lee & Ashton, 2018 |
| I tend to manipulate others to get my way | Machiavellianism | Jonason & Webster, 2010 |
| I have used deceit or lied to get my way. | Machiavellianism | Jonason & Webster, 2010 |
| I have use flattery to get my way | Machiavellianism | Jonason & Webster, 2010 |
| I tend to exploit others towards my own end. | Machiavellianism | Jonason & Webster, 2010 |

*Note.* (r) = reverse-keyed.

**Table S2.** *Correlation matrix of the included variables at the County-level. Including counties with at least 50 respondents per spatial unit.*

| **Variables** | **(1)** | **(2)** | **(3)** | **(4)** | **(5)** | **(6)** | **(7)** | **(8)** | **(9)** | **(10)** | **(11)** | **(12)** | **(13)** | **(14)** |
| --- | --- | --- | --- | --- | --- | --- | --- | --- | --- | --- | --- | --- | --- | --- |
| 1. Debt-to-Income | 1 |  |  |  |  |  |  |  |  |  |  |  |  |  |
| 2. Courage | 0.220^***^ | 1 |  |  |  |  |  |  |  |  |  |  |  |  |
| 3. Openness | -0.00537 | 0.171^***^ | 1 |  |  |  |  |  |  |  |  |  |  |  |
| 4. Agreeableness | -0.00385 | -0.0483 | -0.186^***^ | 1 |  |  |  |  |  |  |  |  |  |  |
| 5. Extraversion | -0.0141 | -0.0973^***^ | -0.219^***^ | 0.353^***^ | 1 |  |  |  |  |  |  |  |  |  |
| 6. Conscientiousness | 0.0637^*^ | 0.108^***^ | -0.0743^**^ | 0.634^***^ | 0.148^***^ | 1 |  |  |  |  |  |  |  |  |
| 7. Neuroticism | -0.0921^**^ | -0.0689^*^ | -0.255^***^ | -0.429^***^ | -0.384^***^ | -0.398^***^ | 1 |  |  |  |  |  |  |  |
| 8. Income | 0.372^***^ | -0.0619^*^ | 0.225^***^ | -0.135^***^ | 0.122^***^ | -0.150^***^ | -0.292^***^ | 1 |  |  |  |  |  |  |
| 9. Age | 0.272^***^ | -0.0309 | 0.0180 | -0.171^***^ | -0.146^***^ | -0.105^***^ | 0.323^***^ | 0.0335 | 1 |  |  |  |  |  |
| 10. Gender | -0.0971^***^ | -0.0802^**^ | 0.176^***^ | 0.275^***^ | 0.0749^**^ | 0.142^***^ | -0.0876^**^ | -0.101^***^ | 0.0657^*^ | 1 |  |  |  |  |
| 11. Unemployment | -0.0566^*^ | 0.137^***^ | 0.0310 | -0.0493 | -0.142^***^ | -0.0384 | 0.183^***^ | -0.320^***^ | 0.0860^**^ | -0.0326 | 1 |  |  |  |
| 12. Poverty | -0.433^***^ | 0.129^***^ | 0.0321 | 0.163^***^ | -0.141^***^ | 0.104^***^ | 0.145^***^ | -0.786^***^ | -0.277^***^ | 0.186^***^ | 0.368^***^ | 1 |  |  |
| 13. Population density | -0.103^***^ | -0.00337 | 0.289^***^ | -0.121^***^ | 0.0167 | -0.0709^*^ | -0.0279 | 0.129^***^ | -0.0833^**^ | 0.148^***^ | 0.0788^**^ | 0.0627^*^ | 1 |  |
| 14. Education | 0.0694^*^ | -0.101^***^ | 0.409^***^ | -0.0661^*^ | 0.177^***^ | -0.110^***^ | -0.444^***^ | 0.715^***^ | -0.149^***^ | 0.0905^**^ | -0.412^***^ | -0.498^***^ | 0.192^***^ | 1 |

*N* = 1,220. ^*^*p* < 0.05, ^**^ *p* < 0.01, ^***^ *p* < 0.001

| **Variables** | **(1)** | **(2)** | **(3)** | **(4)** | **(5)** | **(6)** | **(7)** | **(8)** | **(9)** | **(10)** | **(11)** | **(12)** | **(13)** | **(14)** |
| --- | --- | --- | --- | --- | --- | --- | --- | --- | --- | --- | --- | --- | --- | --- |
| 1. Debt-to-Income | 1 |  |  |  |  |  |  |  |  |  |  |  |  |  |
| 2. Courage | 0.321^***^ | 1 |  |  |  |  |  |  |  |  |  |  |  |  |
| 3. Openness | 0.324^***^ | 0.227^***^ | 1 |  |  |  |  |  |  |  |  |  |  |  |
| 4. Agreeableness | -0.0406 | -0.0430 | -0.262^***^ | 1 |  |  |  |  |  |  |  |  |  |  |
| 5. Extraversion | -0.222^***^ | -0.115^*^ | -0.369^***^ | 0.394^***^ | 1 |  |  |  |  |  |  |  |  |  |
| 6. Conscientiousness | 0.110^*^ | 0.156^**^ | -0.164^**^ | 0.653^***^ | 0.126^*^ | 1 |  |  |  |  |  |  |  |  |
| 7. Neuroticism | -0.257^***^ | -0.102 | -0.143^**^ | -0.476^***^ | -0.282^***^ | -0.393^***^ | 1 |  |  |  |  |  |  |  |
| 8. Income | 0.201^***^ | -0.149^**^ | 0.283^***^ | -0.160^**^ | -0.0321 | -0.231^***^ | -0.202^***^ | 1 |  |  |  |  |  |  |
| 9. Age | 0.160^**^ | -0.0936 | 0.0599 | -0.135^*^ | -0.0570 | 0.0112 | 0.370^***^ | 0.0506 | 1 |  |  |  |  |  |
| 10. Gender | -0.0482 | -0.0489 | 0.122^*^ | 0.331^***^ | 0.125^*^ | 0.216^***^ | -0.0239 | -0.156^**^ | 0.299^***^ | 1 |  |  |  |  |
| 11. Unemployment | 0.135^*^ | 0.227^***^ | 0.0327 | -0.0482 | -0.161^**^ | -0.0874 | 0.0967 | -0.220^***^ | 0.00215 | -0.119^*^ | 1 |  |  |  |
| 12. Poverty | -0.160^**^ | 0.274^***^ | 0.0261 | 0.201^***^ | -0.0351 | 0.130^*^ | 0.0435 | -0.747^***^ | -0.338^***^ | 0.145^**^ | 0.317^***^ | 1 |  |  |
| 13. Population density | -0.0446 | -0.0841 | 0.223^***^ | -0.0399 | 0.119^*^ | -0.105^*^ | -0.0554 | 0.492^***^ | 0.119^*^ | 0.145^**^ | -0.0910 | -0.274^***^ | 1 |  |
| 14. Education | 0.0265 | -0.289^***^ | 0.320^***^ | -0.107^*^ | 0.0627 | -0.200^***^ | -0.305^***^ | 0.642^***^ | -0.163^**^ | -0.0218 | -0.486^***^ | -0.454^***^ | 0.333^***^ | 1 |

**Table S3.** *Correlation matrix of the included variables at the MSA-level. Including MSAs with at least 50 respondents per spatial unit.*

*N* = 352. ^*^ *p* < 0.05, ^**^ *p* < 0.01, ^***^ *p* < 0.001

**Table S4.** *Results of Spatial Regressions Predicting Debt-to-Income at the MSA-level, from Courage (S4a) and Courage + Controls (S4b).*

|  | S4a Courage | | |  |  | S4b Courage + Controls | | |
| --- | --- | --- | --- | --- | --- | --- | --- | --- |
|  | B | *SE* (B) | *z* |  |  | B | *SE* (B) | *z* |
| Predictor |  |  |  |  |  |  |  |  |
| Courage | .26*** | .049 | 5.25 |  |  | .24*** | .051 | 4.69 |
| Controls |  |  |  |  |  |  |  |  |
| Openness | - | - | - |  |  | .11 | .062 | 1.84 |
| Agreeableness | - | - | - |  |  | –.004 | .072 | –..05 |
| Extraversion | - | - | - |  |  | –.16** | .053 | –4.69 |
| Conscientiousness | - | - | - |  |  | .03 | .067 | .48 |
| Neuroticism | - | - | - |  |  | –.30*** | .065 | –4.69 |
| Income | - | - | - |  |  | –.11 | .094 | 1.21 |
| Age | - | - | - |  |  | .30*** | .071 | 4.15 |
| Gender | - | - | - |  |  | –.05 | .054 | –.94 |
| Unemployment | - | - | - |  |  | .11** | .052 | 2.15 |
| Poverty |  |  |  |  |  | –.12* | .087 | –1.36 |
| Population density | - | - | - |  |  | –.16** | .050 | –3.10 |
| Education | - | - | - |  |  | –.01** | .072 | –.11 |
| *R* ^2^ | .13 | | |  |  | .40 | | |

*N* = 352*.* All variables were z-standardized. **p < 0.05. **p < 0.01. ***p < 0.001.*

**Table S5.** *Correlation matrix of the included variables at the State-level. Including States with at least 50 respondents per spatial unit.*

| **Variables** | **(1)** | **(2)** | **(3)** | **(4)** | **(5)** | **(6)** | **(7)** | **(8)** | **(9)** | **(10)** | **(11)** | **(12)** | **(13)** | **(14)** |
| --- | --- | --- | --- | --- | --- | --- | --- | --- | --- | --- | --- | --- | --- | --- |
| 1. Debt-to-Income | 1 |  |  |  |  |  |  |  |  |  |  |  |  |  |
| 2. Courage | 0.489^***^ | 1 |  |  |  |  |  |  |  |  |  |  |  |  |
| 3. Openness | 0.273 | 0.496^***^ | 1 |  |  |  |  |  |  |  |  |  |  |  |
| 4. Agreeableness | 0.156 | -0.0784 | -0.364^*^ | 1 |  |  |  |  |  |  |  |  |  |  |
| 5. Extraversion | -0.326^*^ | -0.376^**^ | -0.221 | 0.358^*^ | 1 |  |  |  |  |  |  |  |  |  |
| 6. Conscientiousness | 0.128 | 0.224 | -0.00296 | 0.663^***^ | 0.318^*^ | 1 |  |  |  |  |  |  |  |  |
| 7. Neuroticism | -0.360^*^ | -0.409^**^ | -0.375^**^ | -0.305^*^ | -0.310^*^ | -0.452^**^ | 1 |  |  |  |  |  |  |  |
| 8. Income | 0.184 | -0.0346 | 0.483^***^ | -0.300^*^ | 0.204 | -0.331^*^ | -0.353^*^ | 1 |  |  |  |  |  |  |
| 9. Age | 0.232 | -0.121 | 0.0999 | -0.137 | -0.324^*^ | -0.296^*^ | 0.473^***^ | 0.00414 | 1 |  |  |  |  |  |
| 10. Gender | -0.0933 | -0.207 | 0.202 | 0.313^*^ | 0.276 | 0.291^*^ | 0.117 | -0.0333 | 0.206 | 1 |  |  |  |  |
| 11. Unemployment | 0.0156 | 0.174 | 0.402^**^ | -0.208 | -0.244 | -0.148 | 0.121 | 0.0423 | 0.298^*^ | 0.193 | 1 |  |  |  |
| 12. Poverty | -0.218 | 0.221 | 0.0416 | 0.227 | -0.118 | 0.421^**^ | 0.119 | -0.710^***^ | -0.212 | 0.352^*^ | 0.276 | 1 |  |  |
| 13. Population density | -0.341^*^ | -0.0835 | 0.605^***^ | -0.310^*^ | 0.335^*^ | 0.0246 | -0.176 | 0.432^**^ | -0.168 | 0.439^**^ | 0.252 | 0.0688 | 1 |  |
| 14. Education | 0.120 | -0.0734 | 0.343^*^ | -0.250 | 0.226 | -0.239 | -0.405^**^ | 0.760^***^ | -0.133 | -0.136 | -0.351^*^ | -0.651^***^ | 0.315^*^ | 1 |

*N* = 49. ^*^ *p* < 0.05, ^**^ *p* < 0.01, ^***^ *p* < 0.001

**Table S6.** *Results of Spatial Regressions Predicting Debt-to-Income at the State-level, from Courage (S6a) and Courage + Controls (S6b).*

|  | S4a Courage | | |  |  | S4b Courage + Controls | | |
| --- | --- | --- | --- | --- | --- | --- | --- | --- |
|  | B | *SE* (B) | *z* |  |  | B | *SE* (B) | *z* |
| Predictor |  |  |  |  |  |  |  |  |
| Courage | .46** | .137 | 3.37 |  |  | .20 | .142 | 1.40 |
| Controls |  |  |  |  |  |  |  |  |
| Openness | - | - | - |  |  | .16 | .298 | .58 |
| Agreeableness | - | - | - |  |  | –.01 | .176 | –.07 |
| Extraversion | - | - | - |  |  | –.15 | .160 | –.96 |
| Conscientiousness | - | - | - |  |  | .11 | .151 | .77 |
| Neuroticism | - | - | - |  |  | –.29 | .194 | –1.49 |
| Income | - | - | - |  |  | .24 | .258 | .93 |
| Age | - | - | - |  |  | .20 | .169 | 1.18 |
| Gender | - | - | - |  |  | .30* | .149 | 2.03 |
| Unemployment | - | - | - |  |  | –.07 | .131 | –.51 |
| Poverty |  |  |  |  |  | –.17 | .253 | –.66 |
| Population density | - | - | - |  |  | –.65** | .204 | –3.19 |
| Education | - | - | - |  |  | –.01** | .180 | –.04 |
| *R* ^2^ | .24 | | |  |  | .66 | | |

*N* = 49*.* All variables were z-standardized. **p < 0.05. **p < 0.01. ***p < 0.001.*

**References**

Gosling, S. D., Rentfrow, P. J., & Swann Jr, W. B. (2003). A very brief measure of the Big-Five personality domains. Journal of Research in Personality, 37(6), 504–528.

Howard, M. C., & Alipour, K. K. (2014). Does the courage measure really measure courage? A theoretical and empirical evaluation. The Journal of Positive Psychology, 9(5), 449–459.

Jonason, P. K., & Webster, G. D. (2010). The dirty dozen: A concise measure of the dark triad. Psychological Assessment, 22(2), 420.

Lee, K., & Ashton, M. C. (2018). Psychometric properties of the HEXACO-100. Assessment, 25(5), 543–556.
